# Resting state functional MRI reveals abnormal network connectivity in orthostatic tremor

**DOI:** 10.1097/MD.0000000000004310

**Published:** 2016-07-22

**Authors:** Julián Benito-León, Elan D. Louis, Eva Manzanedo, Juan Antonio Hernández-Tamames, Juan Álvarez-Linera, José Antonio Molina-Arjona, Michele Matarazzo, Juan Pablo Romero, Cristina Domínguez-González, Ángela Domingo-Santos, Álvaro Sánchez-Ferro

**Affiliations:** a Department of Neurology, University Hospital “12 de Octubre”, Madrid; b Centro de Investigación Biomédica en Red sobre Enfermedades Neurodegenerativas (CIBERNED); c Department of Medicine, Complutense University, Madrid, Spain; d Department of Neurology, Yale School of Medicine; e Department of Chronic Disease Epidemiology, Yale School of Public Health; f Center for Neuroepidemiology and Clinical Neurological Research, Yale School of Medicine and Yale School of Public Health, New Haven, CT, USA; g Neuroimaging Laboratory, Center for Biomedical Technology, Rey Juan Carlos University, Móstoles; h Department of Radiology, Hospital Ruber International; i Faculty of Biosanitary Sciences, Francisco de Vitoria University, Pozuelo de Alarcón, Madrid, Spain; j Research Laboratory of Electronics, Massachusetts Institute of Technology, Cambridge, MA, USA; k Movement Disorders Laboratory, HM CINAC, HM Hospitales, Móstoles (Madrid), Spain.

**Keywords:** case–control study, functional connectivity, magnetic resonance imaging, orthostatic tremor

## Abstract

Very little is known about the pathogenesis of orthostatic tremor (OT). We have observed that OT patients might have deficits in specific aspects of neuropsychological function, particularly those thought to rely on the integrity of the prefrontal cortex, which suggests a possible involvement of frontocerebellar circuits. We examined whether resting-state functional magnetic resonance imaging (fMRI) might provide further insights into the pathogenesis on OT. Resting-state fMRI data in 13 OT patients (11 women and 2 men) and 13 matched healthy controls were analyzed using independent component analysis, in combination with a “dual-regression” technique, to identify group differences in several resting-state networks (RSNs). All participants also underwent neuropsychological testing during the same session. Relative to healthy controls, OT patients showed increased connectivity in RSNs involved in cognitive processes (default mode network [DMN] and frontoparietal networks), and decreased connectivity in the cerebellum and sensorimotor networks. Changes in network integrity were associated not only with duration (DMN and medial visual network), but also with cognitive function. Moreover, in at least 2 networks (DMN and medial visual network), increased connectivity was associated with worse performance on different cognitive domains (attention, executive function, visuospatial ability, visual memory, and language). In this exploratory study, we observed selective impairments of RSNs in OT patients. This and other future resting-state fMRI studies might provide a novel method to understand the pathophysiological mechanisms of motor and nonmotor features of OT.

## 1 Introduction

The term “orthostatic tremor” (OT), also known as “shaky legs syndrome,”^[1]^ was first coined in 1984 by Heilman,^[2]^ although there may have been earlier descriptions of this entity.^[3]^ This is an intriguing and rare condition, characterized by tremor and unsteadiness when standing that is relieved when sitting or walking. OT can be idiopathic or secondary.^[4–6]^ Gerschlager et al^[4]^ suggested the subdivision of OT into 2 broad groups – those with “primary OT” with or without postural arm tremor, and those with “OT plus,” in whom there are additional associated movement disorders, mainly Parkinsonism.

The pathogenesis of OT is poorly understood. Clinical and neuroimaging data suggest that it could arise from a central generator in the cerebellum or brainstem.^[4,6,7]^ Furthermore, we have recently observed that OT patients have deficits in specific aspects of neuropsychological function, particularly those thought to rely on the integrity of the prefrontal cortex, which suggests a possible involvement of frontocerebellar circuits.^[8]^ Notwithstanding, very little is known about the underlying causes and brain networks involved in OT, and further study is needed.

Among various advanced magnetic resonance imaging (MRI) techniques, functional MRI (fMRI) allows one to explore the dynamics of cortical functional reorganization, mainly using activation paradigms evoked by simple motor tasks or cognitive tasks. In task-related fMRI studies, however, there is some difficulty interpreting results due to large intersubject variability in task performance.^[9]^ This limitation of task-related fMRI studies is not a feature of a more recent approach – the acquisition of fMRI data during resting state conditions (i.e., with participants awake, but relaxed and not involved in any task).^[9]^ In this setting, spatially distributed networks of interest can be detected that can characterize resting-state networks (RSNs).^[9]^ These RSNs have demonstrated high reproducibility across participants, time, and research sites, and could serve as surrogate biomarkers for several neurological diseases, including paroxysmal kinesigenic dyskinesia, focal hand dystonia, essential tremor, Alzheimer disease, and Parkinson disease, among others.^[10–14]^

With respect to the motor features of OT, both the cerebellum and sensorimotor networks could be involved. Aside from their possible involvement in these motor symptoms, RSNs alterations might be involved in the pathogenesis of nonmotor manifestations associated with OT. These latter broader networks include the default mode network (DMN) and executive, frontoparietal, auditory/language, and visual networks. However, overall, RSNs integrity in OT patients has not previously been reported.

The present study, using fMRI, compares resting-state functional connectivity in OT patients and healthy controls (HCs) and specifically assessed the following RSNs: DMN, executive network, 2 frontoparietal networks (left- and right-lateralized), as well as sensorimotor, cerebellar, auditory/language, and visual networks. Our a priori study hypotheses were as follows: OT patients will show changes relative to HC in the cerebellar and sensorimotor networks; and several additional RSNs will be impaired in OT patients relative to HC, including the DMN, executive, and the frontoparietal networks (i.e., RSNs that are involved in cognitive processes).^[15,16]^

## 2 Methods

### 2.1 Participants

Patients with OT were consecutively recruited from December 2011 to May 2013 from the outpatient neurology clinics of the University Hospital “12 de Octubre” in Madrid (Spain). Four neurologists, with expertise in movement disorders (JB-L, JPR, MM, and ÁS-F), examined these patients, who were referred to the outpatient neurology clinics with a subjective feeling of unsteadiness when standing, which was absent while walking, seated, or supine. Diagnoses of OT were assigned by the 4 neurologists using the Consensus Statement on Tremor by the Movement Disorder Society.^[17]^

Of 21 eligible OT patients, 7 were excluded from the final cohort because they did not complete the neuropsychological testing or the MRI procedures. Finally, a strict criterion for head movement assessment was adopted (maximal absolute head movement less than 1.0 mm and 1.0° in the x, y, and z directions). One OT woman who failed to meet this criterion was excluded for this reason. No HC was excluded due to incomplete neuropsychological evaluation or refusal to perform MRI.

OT cases were 1:1 frequency-matched with HC. Frequency-matching was based on age, sex, and years of education.

HC were recruited from either relatives or friends of the health professionals working at the University Hospital “12 de Octubre” of Madrid (Spain) or among the relatives of patients who came to the neurological clinics for reasons other than OT (e.g., headache, dizziness). None reported having a 1st- or 2nd-degree relative with OT or essential tremor. Each control was examined by 2 neurologists (JPR and ÁS-F), to further rule out any neurological conditions, and by a neuropsychologist, as noted above.

According to a recently published comorbidity score developed in ambulatory care settings,^[18]^ a comorbidity index was calculated. The presence of several conditions (atrial fibrillation, nonmetastatic cancer, metastatic cancer, chronic obstructive pulmonary disease, depression, dementia, diabetes, epilepsy [treated], heart failure, myocardial infarction, psychiatric disorders, renal disease, and stroke) resulted in the assignment of more points than others, and the score ranged from 0 to 28 (i.e., all conditions present).^[18]^

### 2.2 Neuropsychological testing

All participants underwent a detailed neuropsychological assessment covering the domains of attention, executive function, verbal memory, visual memory, visuospatial ability, and language. These tests have previously been described.^[8]^ No patients were being treated with medication for OT (i.e., clonazepam, dopaminergic agonists, or barbiturates) at the time of the neuropsychological testing because all patients were newly diagnosed at inclusion in the cohort. Neuropsychological tests were conducted in a single session by an experienced clinical neuropsychologist (VP, see acknowledgments) who was blinded to the clinical status during an interview in the week in which the participants completed the below MRI examination.

Raw scores of neuropsychological tests were transformed into z scores based on the mean and standard deviation values from HC. Higher z scores indicated better performance. The severity of depressive symptoms were measured by the original 17-item version of the Hamilton Depression Rating Scale.^[19]^

The tasks from the neuropsychological assessment were z-standardized, averaged, and compiled to create 6 composite scores (attention, executive function, visuospatial ability, verbal memory, visual memory, and language) for each participant. Each composite score was then employed as a continuous variable in subsequent regression analyses.

### 2.3 MRI procedure

Patients and controls were positioned in the scanner and were told to relax with their eyes closed. They were immobilized with a custom-fit blue bag vacuum mold (Medical Intelligence, Schwabmünchen, Germany) to prevent motion artifacts. Earplugs and noise-reduction headphones were used to attenuate scanner noise. The functional run required 6 minutes to complete.

Images were acquired on a General Electric Signa 3T MR Scanner (General Electric Healthcare, Fairfield, CT) using a whole-body radiofrequency coil for signal excitation and quadrature 8-channel coil for reception. Resting-state fMRI data consisted of 120 volumes of a repeated gradient-echo echo planar imaging T2∗-weighted sequence whose parameters were repetition time = 3 seconds, echo time = 28 milliseconds, voxel dimensions = 2.7 × 2.7 × 2.8 mm, 39 oblique ACPC-oriented slices, flip angle = 90°, and 6 dummy scans.

For the structural image, a high-resolution, 3-dimensional T1-weighted gradient Echo-SPGR was acquired with the following parameters: repetition time = 9.2 milliseconds, echo time = 4.128 milliseconds, inversion time = 500 milliseconds, field of view = 240 mm, acquisition matrix = 240 × 240, slice thickness = 1 mm, full brain coverage, resolution = 1 × 1 × 1 mm, flip angle = 120°, and 166 sagittal slices.

### 2.4 Image preprocessing

Resting-state fMRI images were analyzed using FMRIB Software Library (FSL; available at: www.fmrib.ox.ac.uk/fsl) and Analysis of Functional NeuroImages (available at: http://afni.nimh.nih.gov/afni/).^[20,21]^ The preprocessing included the following steps: despiking, slice-timing correction, motion correction, field map correction, spatial smoothing (full-width half maximum = 6 mm), temporal high pass filtering (cut-off of 100 seconds), functional to anatomical image registration, and normalization to the atlas space of the Montreal Neurological Institute (MNI) 152 T1 2 mm template. Despiking was performed using Analysis of Functional NeuroImages, and the remainder of the steps of the preprocessing pipeline were performed with FSL.

### 2.5 Image analysis

Resting-state fMRI data were analyzed using independent component analysis, in combination with a “dual-regression” technique.^[22]^ This method automatically determines the most consistent RSNs, based on an assessment of the similarity of predefined templates.^[22]^

In order to obtain the group independent spatial maps identifying RSNs across all participants, we used the multivariate exploratory linear optimized decomposition into independent components toolbox in FSL. A Temporal Concatenation Group Independent Component Analysis restricting the number of components to 25 was performed to study large-scale spatial networks.^[23]^ Data from all subjects, patients and controls, were concatenated for this analysis.

The 25 independent components were sorted into 2 broad classes: biologically plausible/functionally relevant components or RSNs, and scanner/physiological artifactual components (cerebrospinal fluid, white matter, head motion, and large vessels artifacts). The inspection was made visually based on each component's spatial profile and time course following criteria purposed by Kelly et al.^[24]^ Eight RSNs previously related to functionally relevant brain functions^[25]^ were identified: DMN, executive network, 2 frontoparietal networks (left- and right-lateralized), and sensorimotor, cerebellar, auditory/language, and visual networks.

These 8 independent components spatial maps were used as the RSN spatial map templates in the 1st step of the subsequent dual regression analysis.

The image analysis was performed in 2 steps with FSL-dual regression:^[22]^ each RSN spatial map template was used as a mask in a spatial regression against each individual fMRI dataset in order to obtain a subject specific time course associated to that RSN; and the obtained individual time courses related to each RSN spatial map template in the 1st regression were used in a temporal regression to estimate a subject-specific spatial correlation map per RSN. After this dual regression, spatial maps of all subjects were collected for each original RSN.

Permutation statistics were computed with FSL-randomize to evaluate functional connectivity differences between the 2 groups in each RSN using the previously obtained subject specific spatial maps (number of permutations = 1000). We statistically accounted for effects of age and sex by including these variables as covariates in the statistical model. The dual regression considered the whole brain, not only the areas where each RSN was strongly manifested.^[22]^ Results were considered significant for *P* < 0.005 uncorrected using a threshold-free cluster enhancement.^[26]^ The following information was provided for the clusters whose size was greater than or equal to 10 voxels (80 mm^3^): maximum uncorrected threshold-free cluster enhancement *P* value of the cluster (permutation statistics); cluster size; MNI coordinates of the maximum of the cluster; Talairach atlas label of this region; and the corresponding Brodmann area or the most probable lobule reported in the cerebellar atlas in MNI152 space, after normalization with FMRIB Linear Image Registration Tool.

All procedures were approved by the ethical standards committees on human experimentation at the University Hospital 12 de Octubre (Madrid). Written (signed) informed consent was obtained from all enrollees.

### 2.6 Sample size and statistical analyses of clinical and neuropsychological data

In several recent resting-state fMRI studies of other rare movement disorders, a sample size of 13 to 15 in each group has been sufficient.^[10,11]^

Statistical analyses for the clinical and neuropsychological measures were conducted using SPSS 21 (Statistical Package for the Social Sciences). Mean scores (age and neuropsychological variables) were compared using 2 independent sample *t* tests for continuous and normally distributed data, and Mann–Whitney *U* test for nonnormally distributed data, where appropriate. The χ^2^ test was used to analyze group differences in sex and smoking status.

For the RSNs that were significant after group comparison, the mean z scores of the clusters whose size was greater than or equal to 50 voxels were regressed against disease duration, and each 1 of the 5 different cognitive composite measures, and the 17-item HAMD score. A value of *P* < 0.05 was considered statistically significant.

Failure of any of the test was defined as a z score ≤1.5 standard deviation compared to HC. Cognitive impairment was defined as failure on at least 3 tests.

## 3 Results

### 3.1 Clinical and neuropsychological testing results

All 13 OT patients were right-handed (mean age 65.5, range 37–81). There was a female preponderance (N = 11, 84.6%) with a mean age of onset at 55.9 (range 17–74) years. On diagnosis, 8 (61.5%) of patients presented with primary OT and 5 (38.5%) had additional neurological features (mild parkinsonian signs). Ten (76.9%) patients reported a progressive course. Structural brain MRI was unremarkable in all patients; none had cerebellar atrophy. Routine blood and chemistry tests including thyroid function tests, serum protein electrophoresis, and vitamin B12 levels were also in the normal range in all patients. No patients were being treated with medication for OT (i.e., clonazepam, dopaminergic agonists, gabapentin, or barbiturates) at the time of the neuropsychological testing.

The 13 right-handed OT patients (11 women and 2 men) were compared with 13 right-handed HC (11 women and 2 men). The 13 OT patients did not differ to a significant degree from the 13 HC in terms of age, sex, years of education, comorbidity index, current smoking, and depressive symptoms (Table 1). The results of neuropsychological testing are shown in Table 1. In most domains, OT patients’ cognitive performance was significantly worse than that of the HC. These differences involved selected tests of executive function, visuospatial ability, verbal memory, visual memory, and language (Table 1).

**Table 1:**
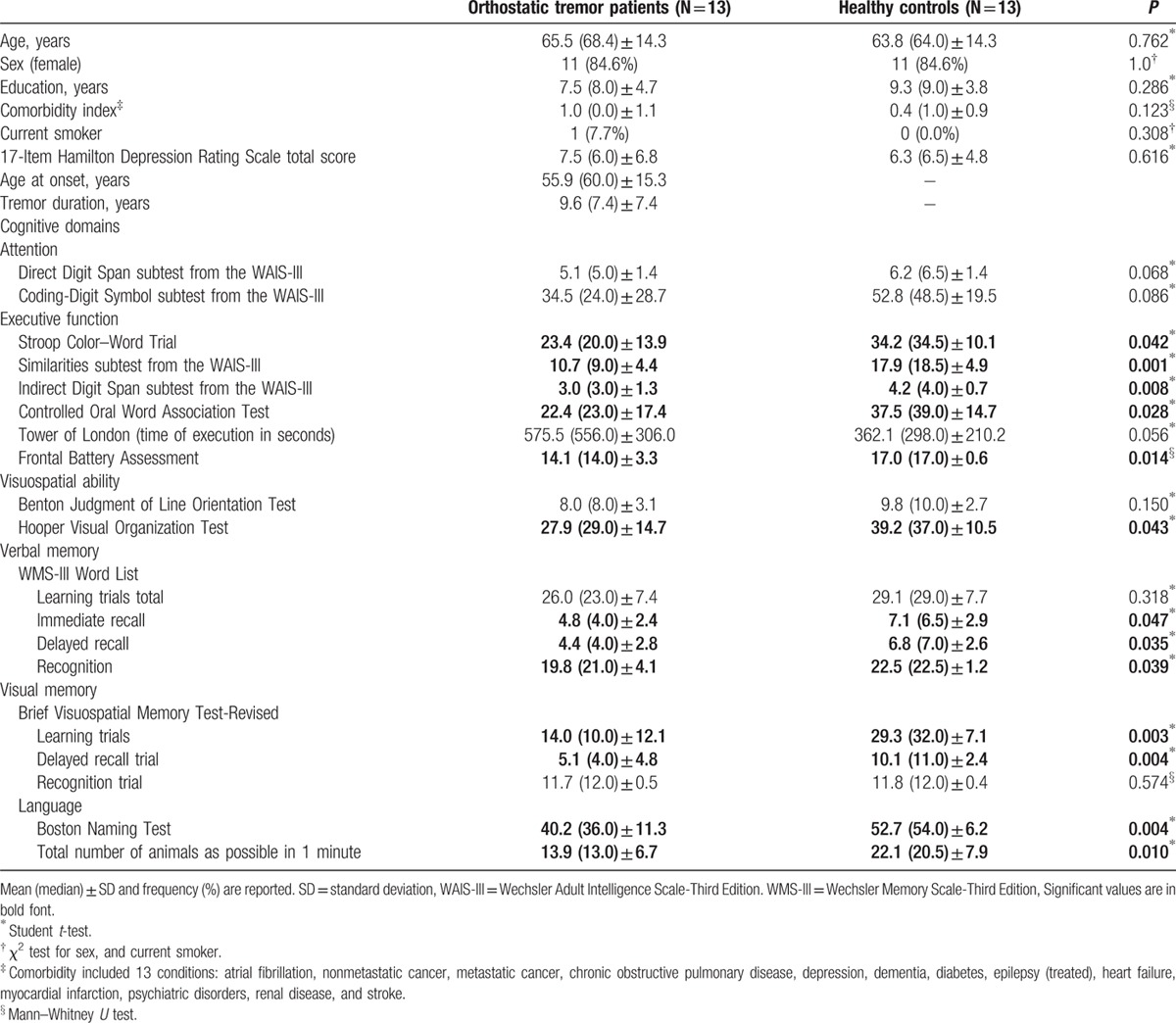
Comparison of demographic, clinical and neuropsychiatric domains of orthostatic tremor patients versus healthy controls.

### 3.2 Resting-state fMRI results

**Figure 1:**
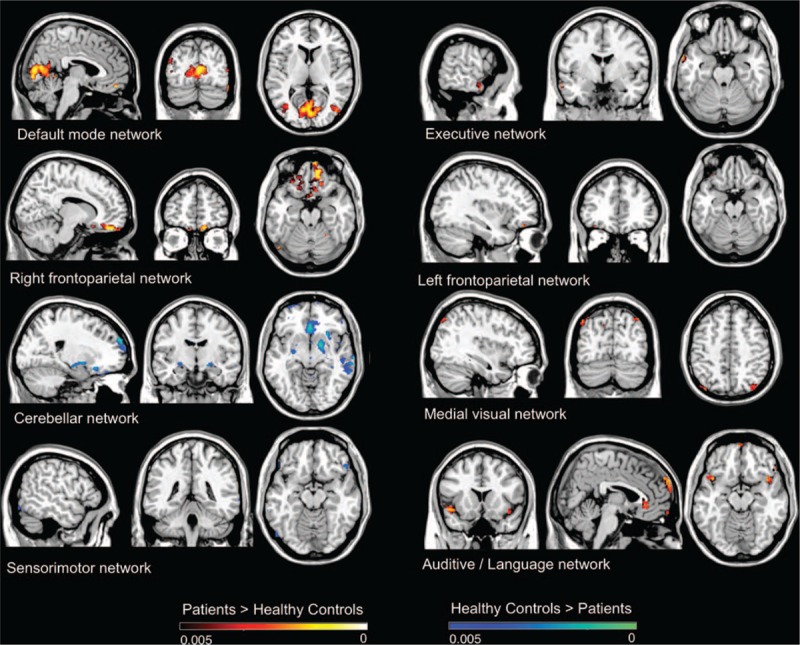
Resting state networks, which showed between-group functional connectivity differences, including Montreal Neurological Institute coordinates and *P*-values for peak voxels of all statistically significant clusters.

All results for the RSNs, which showed between-group functional connectivity differences, including MNI coordinates and *P*-values for peak voxels of all statistically significant clusters, are summarized in Table 2  and visualized in Fig. 1. Overall, OT patients showed changes relative to HC in the cerebellar and sensorimotor networks and in those major RSNs that might be involved in nonmotor symptoms, mainly cognition, including the DMN, executive, and the frontoparietal networks.

**Table 2:**
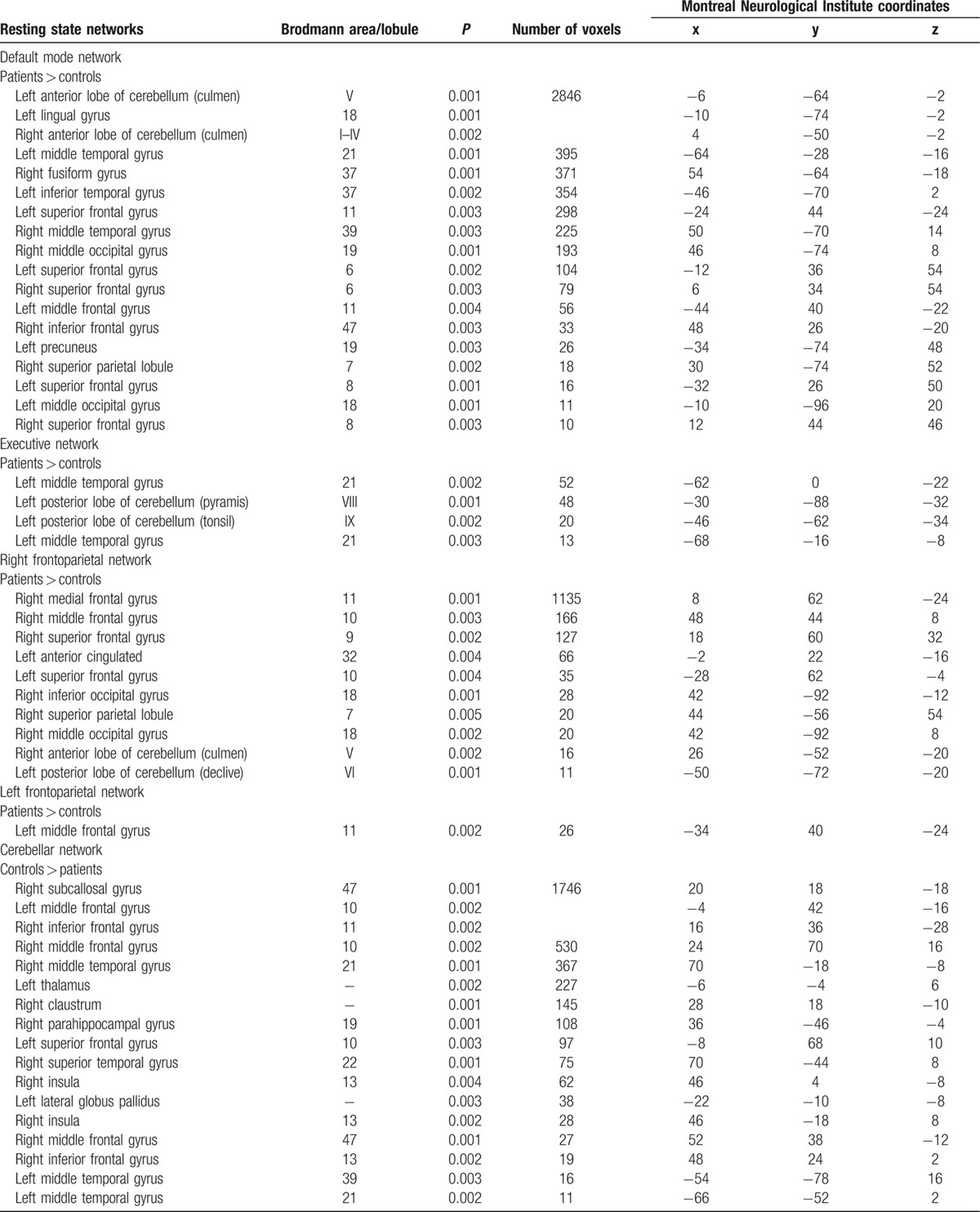
Regions that showed statistically significant differences in functional connectivity between all OT patients versus healthy controls.

**Table 2 (Continued):**
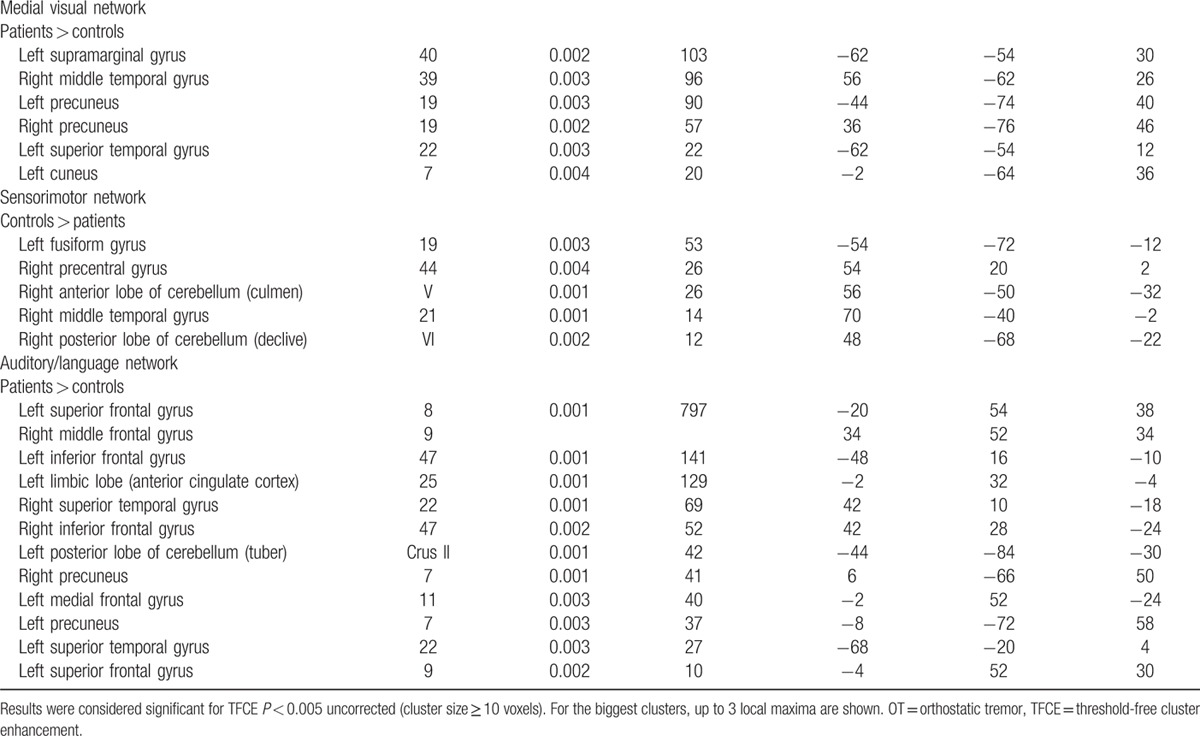
Regions that showed statistically significant differences in functional connectivity between all OT patients versus healthy controls.

In additional analyses, we excluded OT plus cases (N = 5) (i.e., those associated with mild parkinsonian signs on examination), and the results were similar (data not shown). We also excluded 4 OT cases with cognitive impairment (defined as failure on at least 3 tests) (Table 3). In these analyses (Table 3), major RSNs that might be involved in both motor and nonmotor symptoms (i.e., cognition) were altered. However, fewer brain areas were involved in comparison with the analyses that included all OT cases (Table 3).

**Table 3:**
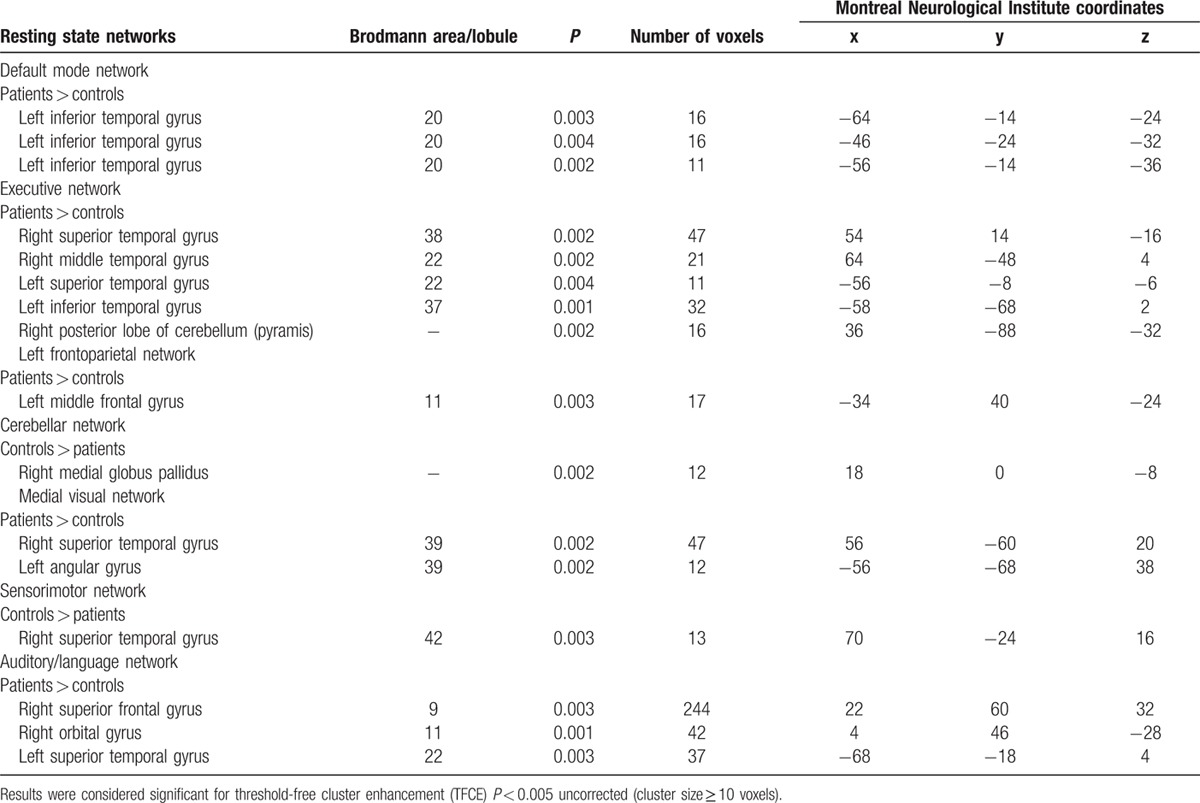
Regions that showed statistically significant differences in functional connectivity in cognitively unimpaired orthostatic tremor patients (N = 9) versus healthy controls.

### 3.3 Relationships between functional connectivity, duration of disease, and cognition

These correlations were calculated in OT patients only, and only for the voxels that showed differences between patients versus HC (see Table 1). There was an association between disease duration and connectivity in the DMN and the medial visual network (Table 4). In addition, connectivity in 2 RSNs (DMN and medial visual network) was associated with cognitive processes (attention, executive function, visuospatial ability, visual memory, and language) (Table 4).

**Table 4:**
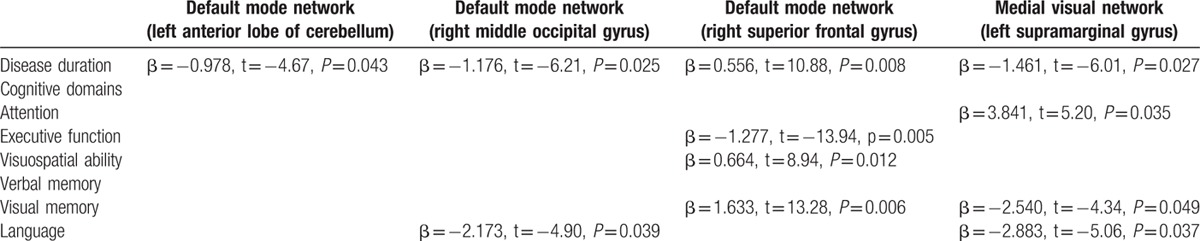
Associations of disease duration and cognitive variables and the mean z value of the significantly differing voxels of functional connectivity in all orthostatic tremor patients.

## 4 Discussion

We investigated functional connectivity in a sample of OT patients and HC. Overall, relative to HC, OT patients showed increased connectivity in RSNs involved in cognitive processes (DMN, as well as in executive and frontoparietal networks), and decreased connectivity in motor control (cerebellum and sensorimotor networks). Changes in network integrity were associated not only with duration (DMN and medial visual network), but also with cognitive function. Moreover, in at least 2 networks (DMN and medial visual network), increased connectivity was associated with worse performance on different cognitive domains (attention, executive function, visuospatial ability, visual memory, and language).

At 1st glance, the presence of increased connectivity seems counterintuitive; this is also found in early multiple sclerosis, mild cognitive impairment, essential tremor, and diabetes.^[27–29]^ In general, RSNs are functionally connected, and dysfunction in 1 network may lead to dysfunction in the other networks.^[30]^ Reduced functional connectivity is thought to reflect dysfunction of the network, and increased functional connectivity has been interpreted as a compensatory mechanism or reorganization of the network.^[30]^

Although the sensorimotor and the visual and auditory networks involve cortical regions normally engaged in sensorimotor, visual, and auditory processes, respectively, the DMN and the executive and the frontoparietal networks are the RSNs most relevant for cognition.^[15,16]^ We found increased functional connectivity in the DMN, as well as executive and frontoparietal networks in OT patients. Of additional interest, we found the right insula to be less connected to the cerebellum network in OT patients. Recent neuroimaging data reveal that the insular cortex is involved in essential tremor or in various neuropsychiatric diseases.^[31,32]^ One may speculate that these insular changes in OT might be an early marker of cognitive impairment in OT. However, this possibility requires further study.

Our results also indicate that OT patients present a certain increased functional connectivity in medial visual and auditory/language networks. The aberrant functional connectivity of both networks found in our study could be associated with perceptual and language impairments in OT patients. In line with this, OT patients scored worse on the Hooper Visual Organization Test,^[33]^ an instrument that measures visual organizational skills,^[33]^ as well as language tests; however, clinical studies have yet to study or document such changes.

Interestingly, major RSNs that might be involved in cognitive function (DMN, executive network, and left frontoparietal network) were altered even in OT patients who were not defined as cognitively impaired. These alterations were, however, subtler than those found when all OT patients were included. We hypothesize that a dysfunction of these RSNs may have a role in the pathogenesis of cognitive dysfunction in OT. Our functional data suggest that there may be an early functional disruption of these RSNs in OT prior to clinical evidence of significant cognitive impairment. This is consistent with evidence in the Alzheimer and Parkinson disease literature, where a functional alteration of the DMN is already present in *APOE*4+ cognitively normal individuals and in cognitively unimpaired Parkinson disease patients, respectively.^[34,35]^ It is also important to note that we defined cognitive impairment conservatively as failure on at least 3 tests rather than failure on 1 or more tests.

The study was not without limitations. First, the sample size was relatively small. The OT literature, however, only includes studies with small sample sizes. One should keep in mind that OT is a very rare disease, and hence it is rather difficult to recruit patients for any case–control study. Although there are no available epidemiological data, in the follow-up evaluation of the Neurological Disorders of Central Spain study,^[36]^ we detected only 1 patient with OT in a cohort of approximately 4000 elderly subjects (data not published). Despite the small sample size, with our sample we could detect a number of differences between the 2 study groups. Second, the recruited sample was quite heterogeneous, including primary and OT plus cases. However, our aim was to examine whether OT patients in general had altered resting state brain networks when compared with matched controls. Furthermore, after exclusion of OT plus cases, the results remained similar. This study also had several strengths. First, this is the first study that has assessed RSN integrity of OT patients. Second, assessments were conducted prospectively in a standardized manner.

In summary, in this exploratory study, we observed selective impairments of RSNs intrinsic functional connectivity in OT patients. This and other future resting-state fMRI studies might provide a novel method to understand the pathophysiological mechanisms of motor and nonmotor features of OT.

## Acknowledgments

The authors thank Dr Verónica Puertas for her assistance with the project.
